# The unmet needs for identifying the ideal bowel preparation

**DOI:** 10.1002/jgh3.12653

**Published:** 2021-09-17

**Authors:** Gian E Tontini, Alberto Prada, Sandro Sferrazza, Giorgio Ciprandi, Maurizio Vecchi

**Affiliations:** ^1^ Department of Internal Medicine Gastroenterology and Endoscopy Unit, Fondazione IRCCS Ca' Granda Ospedale Maggiore Policlinico Milan Italy; ^2^ Digestive Endoscopy Istituto Auxologico Italiano Milan Italy; ^3^ Gastroenterology and Endoscopy Unit, Area Chirurgia Specialistica Santa Chiara Hospital Trento Italy; ^4^ Outpatient Department, Casa di Cura Villa Montallegro Genoa Italy

**Keywords:** bowel preparation, colonoscopy, patient satisfaction

## Abstract

Colonoscopy, since it was first employed over 60 years ago, is now the gold standard method for visualizing the mucosa of the colon, but should be of good quality. Many factors affect quality, including the type of health service organization, type of facility, staff, equipment, patient characteristics, and bowel preparation (BP). The adequacy of bowel cleansing is critical, but, unfortunately, may be inadequate in up to one‐third of procedures. The current article will present and discuss the main BPs and their drawbacks, which include patient‐dependent and procedure‐dependent factors. Cleansing quality depends on the ease/complexity of solution preparation, volume, taste, and timing of consumption. Consequently, important positive factors include simple instructions, easy preparation of the solution, low volume, pleasant taste, short drinking time (e.g. <30 min), and splitting the dose between the evening before and the morning of the colonoscopy (or even better, only one dose in the early morning to avoid night‐time problems), and short onset of action. The BP solution must also be safe with negligible side effects. Furthermore, a positive experience supports patient willingness to repeat the procedure.

## Graphical abstract

The present bowel preparations (BPs) present a series of drawbacks. The ideal BP should be effective, safe, easily self‐administered, have good patient acceptance, and be low cost.

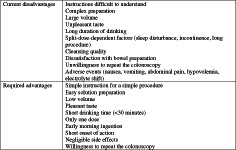

## Introduction

Colonoscopy, since it was first employed over 60 years ago, has become the gold standard method for visualizing the mucosa of the colon. Some 20 million such procedures are performed in Europe and the United States annually.^1^ The main indications for colonoscopy are screening, diagnosis confirmation, and surveillance, as summarized in Table 1.^2^ The demand for colonoscopy has doubled in the last few years because of the aging of the general population and the implementation of bowel cancer screening programs.^3^ Good quality colonoscopy is essential,^4^ but unfortunately, up to one‐third of procedures may be of inadequate quality and so of little use.^5^, ^6^

**Table 1:** Indications for colonoscopy

Screening	Colorectal cancer screening programs
	Subjects with a family history of colorectal cancer
Diagnosis	Gastrointestinal bleeding
	Unexplained weight loss
	Sideropenic anemia
	Persistent and relevant change in bowel movements
	Unexplained abdominal pain
Confirmation	Colorectal alterations previously detected by barium enema, computed tomography, or magnetic resonance
	After virtual colonoscopy
Surveillance	Patients with intestinal adenoma and/or polyps
	Patients with chronic inflammatory intestinal disease
	Follow‐up after bowel cancer surgery

Many factors affect colonoscopy quality, including the type of health service organization, type of facility, staff, equipment, patient characteristics, and the bowel preparation (BP) process (Table 2). High‐quality colonoscopy is essential for successful screening programs and effective diagnosis.^7^, ^8^ The BP process is especially important.^9^, ^10^, ^11^ Inadequate BP results in a lengthy procedure, incomplete colonoscopy, incorrect diagnosis or diagnostic delay, increased costs due to the need to repeat the procedure, adverse events, and patient unwillingness to repeat the examination.^12^, ^13^, ^14^, ^15^ BP is inadequate in 18–35% of colonoscopies.^16^ The main factors affecting the BP procedure are the patient's characteristics and the BP reconstitution/consumption process, as reported in Table 3.

**Table 2:** Factors affecting the quality of colonoscopy

Health service organization	Public hospital
	Private insurance
	Open access
Facility	Inpatient
	Outpatient
Staff	Operator (ability, attitude)
	Nursing (communication)
Equipment	Endoscope reliability
Bowel preparation	Purgatives
	Medications
	Diet
	Timing
	Instructions
	Arrangement
Patient	Age
	Sex
	Education
	Cognitive status
	Health literacy
	Fear
	Humiliation
	Embarrassment
	Modesty
	Depression
	Adherence
	Tolerability
	Palatability
	Adverse event experience

**Table 3:** Factors associated with quality of bowel preparation

Patient‐dependent factors	Motivation
	Instruction
	Health literacy
	Education
	Socioeconomic status
	Adherence
	Comorbidities and hospitalization
	Discomfort
Product‐dependent factors	Volume
	Mechanism of action
	Osmolarity
	Taste
	Dosing
	Complexity of the split schedule
	Timing
	Runway time
	Safety (adverse events, including intestinal gas)
	Tolerability

Various guidelines and recommendations have been published.^12^, ^13^, ^14^, ^15^, ^16^, ^17^, ^18^, ^19^, ^20^, ^21^ Over 90% of procedures should meet a minimum standard (measured by validated scales), as recently recommended by the European Society of Gastrointestinal Endoscopy (ESGE).^15^ The American Society for Gastroenterology Endoscopy (ASGE) has stated that BP should effectively clear the colon of stool and provide maximal visualization of mucosa, preserve the gross and microscopic integrity of the colon, and be easily administered, well tolerated, inexpensive, and safe, avoiding shifts in fluids and electrolytes.^18^ Remarkably, none of the currently available BP products meet all of these requirements. This article will analyze and discuss the various aspects of BP in clinical practice. Currently available BP formulations will be described, and unmet needs will be discussed in order to identify the ideal preparation as envisaged in the literature.^22^, ^23^, ^24^, ^25^, ^26^

## BP quality assessment

A good quality colonoscopy is required to identify colonic lesions and, if possible, treat them, as in screening programs for colorectal cancer.^27^, ^28^ Rex *et al*. described in detail the requirements for good quality colonoscopy.^1^ Therefore, it is clinically important to assess BP quality^29^ and several scales are used, including the Aronchick Scale, Boston Bowel Preparation Scale (BBPS), Chicago Bowel Preparation Scale, Harefield Cleansing Scale, Marden Bowel Preparation Classification, and Ottawa Bowel Preparation Scale (OBPS), as reported in Table 4.^30^, ^31^ Each of these scales has different advantages, so the endoscopist picks one based on personal experience, local protocols, and guidelines. However, the OBPS and the BBPS are the most commonly used in clinical practice. The OBPS is preferred for evaluating the efficacy of preparation before intervention, while the BBPS is used to evaluate the efficacy of preparation after aspiration and lavage, hereby measuring the ability to visualize the mucosa and the real diagnostic reliability of each colonoscopy.

**Table 4:** Bowel preparation scales available in clinical practice

Scale	Scale range	Excellent score	Inadequate score
Aronchick Score			
Ottawa Bowel Preparation Scale	0–14	0	14
Boston Bowel Preparation Scale	0–9	9	0
Harefield Cleansing Scale	0–20	20	0
Chicago Bowel Preparation Scale	0–36	36	0
Marden Bowel Preparation Classification	1–4	4	1

## BP products

BP products can be mainly classified into two broad categories: polyethylene glycol (PEG)‐based products and hyperosmotic products (Table 5). Both types can produce adequate bowel cleansing, but with variable tolerance, preparation‐induced mucosal changes, and adverse events.

**Table 5:** Bowel preparation products: main characteristics

BP products	Total volume ingested	Regimen	BP efficacy (adequate colonoscopies)	ADR	PDR	Indication	Limitations	Adverse events (most common)
PEG‐ELS	4 L	Split	71–92%	27–34%		Preferred in patients with IBD, renal failure. Liver disease Consider in elderly and inpatients	High volume and side effects	Abdominal pain, bloating, nausea, vomiting, incontinence
PEG‐ELS + ascorbic acid	2.95 L	Split	74–93%	18–25%		Consider in otherwise healthy outpatients	Avoid in patients with G6PD deficiency	Nausea, gastralgia, vomiting, dizziness, dehydration
PEG 3350 without ELS	1.9 L	Split	67–81%		47%	Consider in young people	Avoid in patients with severe constipation and in patients with hemorrhage as the onset of action is slow	Nausea, abdominal fullness, bloating
Magnesium citrate	2.3 L	Split	75–90%			Constipation	Elderly patients and patients with nephropathy, congestive heart failure, hypermagnesemia. Additional water drinking could be advised in some patients with the evening dose	Nausea, vomiting, abdominal pain. Electrolyte imbalance. Dehydration. Headache
Sodium phosphate	1.6 L	Split	84–90%	27–36%	54%	Effective in patients with constipation	Avoid in elderly patients, concomitant use of NSAID and ACEi for nephrotoxicity, and patients with suspected IBD	Abdominal bloating, nausea, abdominal pain, vomiting. Phosphate nephropathy (rare)
Sodium sulfate	2.84 L	Split	93–98%	26%	50%	Alternative to sodium phosphate	Avoid in gout (possible increase of serum uric acid)	Overall discomfort, abdominal distension, abdominal pain, nausea, vomiting, headache
Sodium picosulfate	2.2 L	Split	81–88%	23–31%	38–42%	Consider in patients with gastric resection (low volume)	Avoid in elderly patients because of the risk of hyponatremia.	Nausea, vomiting, headache

ADR, adenoma detection rate; BP, bowel preparation; ELS, electrolyte lavage solution; IBD, inflammatory bowel disease; PDR, polyp detection rate.

PEG is a polymer prepared by the polymerization of ethylene oxide and is widely used in medicine and vaccine preparation. Standard PEG‐based BP involves the consumption of 2–4 L of solution in 2 h. PEG usually causes significant fluid and/or electrolyte shifts that offset each other to minimize their effect.

A variety of PEG‐based formulations are available, which differ regarding solution volume, electrolyte content, requirement for an adjuvant laxative, the addition of oral simethicone, and the presence of artificial sweeteners. They are usually safe but can have uncommon, but potentially serious, adverse effects, including electrolyte disturbance, allergic reaction, and renal failure.^32^

Hyperosmotic preparations contain poorly absorbed multivalent cations or anions with osmotic effects and increase intraluminal water, causing bowel distension and evacuation. These agents include sodium phosphate (NaP), sodium picosulfate, and magnesium citrate (MC).

NaP preparations are effective and better tolerated than PEG‐based preparations because of their low volume.^33^ However, NaP can have adverse effects including fluid shifts, hyperphosphatemia, electrolyte abnormalities, tonic–clonic seizure, mucosal damage, and acute renal failure, such as acute phosphate nephropathy.^34^ Indeed, the Food and Drug Administration (FDA) has issued a black box warning because of the nephropathy, and NaP as a BP has been removed from the US market.

MC is a hyperosmotic laxative but is not approved by the FDA for BP.

A new dual‐action hyperosmotic preparation contains sodium picosulfate (stimulant laxative) and magnesium (osmotic agent). It is better tolerated than PEG‐based formulations with a similar degree of bowel cleansing, but can precipitate severe hyponatremia in older adults.

## BP volume

An obstacle to successful BP is the large volume of reconstituted solution. Patients drinking 4 L of PEG‐based formulation frequently experience cramps, fullness, nausea, and/or vomiting due to the unpleasant sulfate taste; consequently, about 15% of patients do not complete BP.^35^ However, low‐volume PEG and sulfate‐free solutions (2 L) have now been developed. A further option is to split the preparation volume into two doses so that half of the dose (1 L only) is taken the night before and the other half (1 L) on the day of the procedure. There is overwhelming evidence that a split‐dose regimen is better than a single dose given the day before, as demonstrated by three meta‐analyses.^36^, ^37^, ^38^ A recent meta‐analysis, including 17 randomized controlled trials, also showed that a low‐volume (≤2 L) split‐dose regimen is as effective as a high‐volume split‐dose regimen for cleansing but is better tolerated and has superior compliance.^23^

Same‐day preparation (early in the morning) is used for afternoon colonoscopy. Several studies have showed no substantial differences between split‐dose and same‐day schedules.^39^, ^40^, ^41^, ^42^ However, the schedule chosen should be based on the patient's clinical characteristics, including comorbidities, concomitant medications, and hospitalization, and socioeconomic status.^41^, ^43^

Recently, a 1‐L PEG‐based BP (with ascorbic acid, sodium aspartate, and sulfate) was found to be as effective for colon cleansing as a regular PEG split‐dose regimen.^44^ This combined product has been approved by the FDA for BP for colonoscopy.^45^ A small meta‐analysis confirmed efficacy and safety.^46^

## BP timing

Another critical issue is the interval between consumption of the last dose of preparation solution and the beginning of colonoscopy (“runway time”), as outlined by a recent study conducted in 3205 patients.^47^ An inverse correlation was consistently found between mucosal cleanliness and runway time; the optimal time for the last dose is 3 h before colonoscopy as recommended by the ESGE guidelines.^15^ The same considerations apply to the consumption of a clear liquid diet. Thus, according to the American Society of Anesthesiologists, 2 h is the minimum interval between any ingestion and the procedure.^48^

## Adjuvant drugs

Bisacodyl, a diphenylmethane derivative and stimulant laxative, has been available as a laxative since 1952. It has dual activity in the colon, an anti‐absorptive‐secretory effect and a direct prokinetic effect, through stimulation of parasympathetic nerve endings in the colonic mucosa.^49^ It acts locally in the large bowel by stimulating myoelectrical and motor activity and intestinal secretion, thus enhancing colon motility, reducing overall colonic transit time, and increasing the water content of stool. For these reasons, it has been successfully combined with 2‐L PEG with improved tolerability.^15^, ^50^

Prokinetics are also used to reduce the laxative dose. Mosapride citrate, a selective 5‐hydroxytryptamine‐4 receptor agonist, when used with a split dose of PEG plus ascorbic acid, increased BP efficacy in elderly patients and reduced adverse events, mainly abdominal fullness.^51^

A meta‐analysis showed that prucalopride, a 5‐hydroxytryptamine‐4 receptor agonist that stimulates gastrointestinal peristalsis, when combined with low‐volume BP was as effective as standard low‐volume BP solutions but had fewer adverse events.^52^ However, conclusive evidence is lacking, so the ESGE guidelines do not recommend the routine use of prokinetic medications for BP.^15^

## Simethicone

Simethicone is a silicon dioxide derivative used as an antifoaming agent to reduce bloating, discomfort, or pain caused by excessive gas. The presence of foam is a disturbing element during colonoscopy as it reduces endoscopic visibility and makes additive washing maneuvers necessary. There is evidence that oral simethicone reduces gas volume, as documented by four randomized controlled trials, and improves bowel cleanliness, as demonstrated by randomized controlled trials.^53^ Consequently, its use is recommended by the ESGE guidelines,^15^ and several simethicone‐added PEG formulations are available worldwide.

## Diet

A low‐residue diet, for instance containing <10 g fiber per day, or a clear liquid diet are recommended for BP. According to a recent meta‐analysis, the low residue/regular diet is associated with higher willingness to repeat the colonoscopy (relative risk [RR] 1.08, 95% confidence interval [CI] 1.01–1.16), better tolerability (RR 1.04, 95% CI 1.01–1.08), and adherence (RR 1.04, 95 % CI 1.01–1.08) when compared with a clear liquid diet.^54^ Traditionally, dietary restriction was recommended for 3 days before colonoscopy, but the acceptance rate was very low. A recent study confirmed a previous meta‐analysis, showing that a 3‐day low‐residue diet did not result in better BP quality than a 1‐day diet.^55^ As a result, the ESGE guidelines recommend a low‐fiber diet on the day preceding colonoscopy.^15^

## Instructions

BP education is essential for achieving quality colonoscopy. Nursing staff should provide oral and written instructions, but they are often supplied by other staff or even sent by email. Inadequate instruction can result in misunderstanding that, in turn, discourages colonoscopy uptake.^56^ Thus, the use of enhanced instructions for BP is recommended.^15^ Indeed, a meta‐analysis of 18 randomized controlled trials (with 6536 patients) confirmed that better education improves BP quality. Reminder systems based on automated time‐released text message and/or telephone call to patients who are due for colonoscopy examinations can also significantly improve adherence, BP quality, and adenoma detection^57^, ^58^, ^59^ (X).

## Patient factors

Many patient‐associated factors can affect BP quality. The most important predictor of inadequate preparation is previous inadequate preparation.^14^ Age, sex, physical activity, socioeconomic status, educational level, and comorbidity may significantly affect colonoscopy quality.^1^, ^60^, ^61^ Predictors of poor BP include age (odds ratio [OR] = 1.14), tobacco use (OR = 1.28), narcotic use (OR = 1.28), hypertension (OR = 1.25), diabetes mellitus (OR = 1.38), obesity (OR = 1.46), low education (OR = 1.49), dementia (OR = 2.09), and calcium‐channel blockers (OR = 3.2), as reported in different reviews and meta‐analyses.^61^, ^62^, ^63^, ^64^ Hospitalization is another independent predictor of poor BP.^65^ A recent Italian multicenter study developed and validated a model to identify hospitalized patients with inadequate bowel cleansing.^66^

## Patient acceptance

Different dosing regimens, timing, and preparations have been investigated for improving patient acceptance of BP.^46^, ^67^, ^68^, ^69^, ^70^, ^71^, ^72^, ^73^, ^74^ Low volume PEG (*vs* high volume PEG) and split‐dose or same‐day regimens (*vs* day‐before regimens) have been usually shown to achieve higher acceptance rate and willing to repeat the endoscopic procedure.^15^ However, the ideal regimen has not yet been defined. Coskun and Yuksel proposed a split high‐dose (1 g) sennoside solution as an alternative to 4 L of PEG as patients taking senna experienced less vomiting and nausea, but they did have more abdominal pain.^75^

## Conclusions

BP quality is influenced by patient and procedural factors. As a rule, low adherence and/or poor tolerance of BP significantly affect outcome, even though one study found that less than 20% of patients with inadequate colon preparation reported failure to adequately follow preparation instructions.^76^ The lack of efficacy of a BP protocol also depends on patient diet and the cleansing products used. Timing, including runway time, seems to be crucial. The type of administration (full or split‐dose) is likewise important. In particular, the split‐dose regimen has shown several advantages in terms of colon cleansing and patient tolerability, but it also involves patient inconvenience, sleep disruption, and incontinence.

Personalized medicine is being increasingly implemented in clinical practice. The characteristics of the patient, including their comorbidities, emotional status, education, and socioeconomic level, can affect BP acceptance and efficacy. The most important issue is adherence to the BP process, so detailed discussion with the patient regarding BP is crucial. Patient engagement is also essential for successful BP^77^ and so should be multidisciplinary, involving all staff. Shared responsibility between the clinician and the patient is similarly important for achieving optimal BP and involves selecting the type of preparation for each patient and the provision of clear instructions to optimize patient understanding and compliance.

BP procedures also have some shortcomings.^78^ These include the complexity of solution preparation, large volume, unpleasant taste, long duration of drinking (possibly >1 h), cleansing quality, and splitting schedule. Moreover, adverse events can occur, with a very few being serious.

Consequently, the BP procedure needs to be improved (Table 6). This includes the provision of simple instructions for a simple procedure, easy solution preparation, low volume, pleasant taste, short drinking time (e.g. <30 min), only one dose in the early morning, so avoiding night‐time problems, and short onset of action. From an organizational perspective, if the patient could consume the solution in the early morning, the procedure could be performed in the late morning. Products with negligible side effects are also important as a positive experience will make the patient more willing to repeat the procedure.

**Table 6:** Advantages and disadvantages of current bowel preparation formulations

Current disadvantages	Instructions difficult to understand
	Complex preparation
	Large volume
	Unpleasant taste
	Long duration of drinking
	Split‐dose‐dependent factors (sleep disturbance, incontinence, long procedure)
	Cleansing quality
	Dissatisfaction with bowel preparation
	Unwillingness to repeat the colonoscopy
	Adverse events (nausea, vomiting, abdominal pain, hypovolemia, electrolyte shift)
Required advantages	Simple instruction for a simple procedure
	Easy solution preparation
	Low volume
	Pleasant taste
	Short drinking time (<30 min)
	Only one dose
	Early morning ingestion
	Short onset of action
	Negligible side effects
	Willingness to repeat the colonoscopy

In conclusion, the ideal BP for colonoscopy should be effective, safe, easily self‐administered; have good patient acceptance; and be of low cost. Although currently available preparations are reasonably effective and safe, patient acceptance can still be improved, so further studies are necessary.
